# Lithocholic Acid, a Metabolite of the Microbiome, Increases Oxidative Stress in Breast Cancer

**DOI:** 10.3390/cancers11091255

**Published:** 2019-08-27

**Authors:** Patrik Kovács, Tamás Csonka, Tünde Kovács, Zsanett Sári, Gyula Ujlaki, Adrien Sipos, Zsolt Karányi, Dóra Szeőcs, Csaba Hegedűs, Karen Uray, Laura Jankó, Máté Kiss, Borbála Kiss, Damya Laoui, László Virág, Gábor Méhes, Péter Bai, Edit Mikó

**Affiliations:** 1 Departments of Medical Chemistry, Faculty of Medicine, University of Debrecen, 4032 Debrecen, Hungary; 2 Departments of Pathology, Faculty of Medicine, University of Debrecen, 4032 Debrecen, Hungary; 3 Departments of Internal Medicine, Faculty of Medicine, University of Debrecen, 4032 Debrecen, Hungary; 4 Laboratory of Cellular and Molecular Immunology, Vrije Universiteit Brussel, 1050 Brussels, Belgium; 5 Laboratory of Myeloid Cell Immunology, VIB Center for Inflammation Research, 1050 Brussels, Belgium; 6 Departments of Dermatology, Faculty of Medicine, University of Debrecen, 4032 Debrecen, Hungary; 7 MTA-DE Lendület Laboratory of Cellular Metabolism, 4032 Debrecen, Hungary; 8 Research Center for Molecular Medicine, Faculty of Medicine, University of Debrecen, 4032 Debrecen, Hungary

**Keywords:** lithocholic acid, oxidative stress, breast cancer, NRF2, iNOS, peroxynitrite, 4HNE

## Abstract

In breast cancer patients, the diversity of the microbiome decreases, coinciding with decreased production of cytostatic bacterial metabolites like lithocholic acid (LCA). We hypothesized that LCA can modulate oxidative stress to exert cytostatic effects in breast cancer cells. Treatment of breast cancer cells with LCA decreased nuclear factor-2 (NRF2) expression and increased Kelch-like ECH associating protein 1 (KEAP1) expression via activation of Takeda G-protein coupled receptor (TGR5) and constitutive androstane receptor (CAR). Altered NRF2 and KEAP1 expression subsequently led to decreased expression of glutathione peroxidase 3 (GPX3), an antioxidant enzyme, and increased expression of inducible nitric oxide synthase (iNOS). The imbalance between the pro- and antioxidant enzymes increased cytostatic effects via increased levels of lipid and protein oxidation. These effects were reversed by the pharmacological induction of NRF2 with RA839, tBHQ, or by thiol antioxidants. The expression of key components of the LCA-elicited cytostatic pathway (iNOS and 4HNE) gradually decreased as the breast cancer stage advanced. The level of lipid peroxidation in tumors negatively correlated with the mitotic index. The overexpression of iNOS, nNOS, CAR, KEAP1, NOX4, and TGR5 or the downregulation of NRF2 correlated with better survival in breast cancer patients, except for triple negative cases. Taken together, LCA, a metabolite of the gut microbiome, elicits oxidative stress that slows down the proliferation of breast cancer cells. The LCA–oxidative stress protective pathway is lost as breast cancer progresses, and the loss correlates with poor prognosis.

## 1. Introduction

Evidence is accumulating for the role of bacterial dysbiosis in the pathogenesis of different cancers [1,2,3,4,5,6,7,8,9,10,11,12,13,14,15,16]. Bacteria secrete metabolites that either exert their effects locally, in a paracrine fashion, or enter the circulation and modulate distantly located cancer cells. For paracrine metabolites, the best examples are those involved in the carcinogenesis of colorectal cancer [17]. The metabolites that act on distantly located cancer cells have similar properties as classical human hormones; they are produced in a “gland”, i.e., the microbiome, and then transported to distant organs, where they regulate physiology and behavior [16]. Cadaverine [18], lithocholic acid (LCA) [19], deoxycholic acid [2], and short chain fatty acids [20] have been identified as hormone-like metabolites. These metabolites have pleiotropic effects and modulate multiple cancer hallmarks simultaneously [2,18,19]. The metabolites can inhibit proliferation, decrease epithelial-to-mesenchymal transition, reduce tumor metastasis, decrease cell migration and transmigration, induce antitumor immunity, rearrange cellular metabolism, induce senescence, and reduce cancer cell stemness [2,18,19,21].

LCA, in particular, inhibits the proliferation of breast cancer cells [19,22,23,24,25,26]. Serum glycolithocholate sulfate levels negatively correlate with the proliferation marker Ki67 in human breast cancers (Reference [24] additional file 9, line 110). Moreover, the bacterial machinery for LCA biosynthesis is suppressed in the early stages of breast cancer [19]. LCA has cytostatic properties that are specific to transformed cells [19,22,25]. LCA exerts its anticancer effects through the Takeda G-protein coupled receptor (TGR5) [19].

Breast cancer is a heterogeneous disease and there are well-established systems for the classification of breast cancer cases. The AJCC TNM classification [27,28] is based on the size of the primary tumor, presence of tumor cells in draining lymph nodes, and the existence of distant metastases. Stage 0 reflects in situ carcinoma, while increasing stages (I–IV) denote the spreading of the disease. The Nottingham grading system (grade I–III) [29,30] is used to assess the aggressiveness of the disease, where a higher grade reflects worse clinical outcomes. Finally, the molecular subtypes of breast cancer [31] are based on the driver gene mutations, which coincide with the expression of pharmacological targets in the tumors. The following molecular subtypes exist for breast cancer: Luminal A (ER+, HER2−, Ki67^low^, PgR^high^), Luminal B (ER+, HER2−, either Ki67^high^ or PgR^low^ or ER+, HER2+, any Ki67, any PgR), Her2+ (HER2+, ER−, and PgR−), and triple negative cases (TNBC, HER2−, ER−, PgR−) [31].

Oxidative stress evasion is critical in cancers [32,33]. Thus, antioxidant defense systems, driven by nuclear factor erythroid 2-related factor 2 (NRF2), play a crucial role in supporting breast cancer progression [24,34,35,36]. LCA is associated with the induction of oxidative stress with a preference towards lipid modifications [37,38]. Moreover, LCA treatment modulates NRF2 activity and expression in model systems other than breast cancer [39,40]. We hypothesized that LCA can induce oxidative stress to exert cytostatic effects in breast cancer cells.

## 2. Materials and Methods

### 2.1. Chemicals

All chemicals were from Sigma-Aldrich unless otherwise stated. Lithocholic acid (LCA), cholic acid (CA), chenodeoxycholic acid (CDCA), glutathione (GSH), N-acetyl-cysteine (NAC), and tert-butylhydrquinone (tBHQ) were from Sigma-Aldrich (St. Louis, MO, USA). LCA was used at concentrations of 0.1 µm, 0.3 µm, and 1 µm, which corresponded to LCA concentrations in the breast [41]. GSH and NAC antioxidants were used at a final concentration of 5 mm. The NRF2 activator, tBHQ, was used at concentrations of 5 µm and 10 µm. TGR5 downstream signaling was inhibited using NF449 (a Gsα-selective antagonist). To inhibit nuclear receptor activation CINPA1 (CAR receptor antagonist), DY268 (FXR receptor antagonist), GSK2033 (LXR receptor antagonist)) were used. RA839, an NRF2 activator, which were obtained from Tocris Bioscience (Bristol, UK) and were used at a final concentrations of 5 μm and/or 10 µm. The proteasome inhibitor MG-132 was obtained from Calbiochem and was used at concentrations of 50 nM and 100 nM. The Silencer Select siRNAs targeting TGR5 (GPBAR1—siRNA ID: #1 s195791, #2 s45559, #3 s45558), CAR (NR1I3—siRNA ID: #1 s19369, #2 s19370, #3 s19368), VDR (siRNA ID: s14777), PXR (NR1I2—siRNA ID: s16910), and NRF2 (siRNA ID: #1 s9493, #2 s9492, #3 9491) and the negative control siRNA #1 (cat.no. 4390843) were obtained from Thermo Fisher Scientific and were used at a final concentration of 30 nM.

### 2.2. Cell Lines

The 4T1 cells were maintained in RPMI-1640 (Sigma-Aldrich) medium containing 10% FBS and 1% penicillin/streptomycin, 2 mm L-glutamine, and 1% pyruvate at 37 °C with 5% CO_2_. The MCF7 cells were maintained in MEM (Sigma-Aldrich) medium supplemented with 10% FBS, 1% penicillin/streptomycin, and 2 mm L-glutamine at 37 °C with 5% CO_2_. The SKBR3 cells were maintained in DMEM (Sigma-Aldrich, 1000 mg/L glucose) medium supplemented with 10% FBS, 1% penicillin/streptomycin, and 2 mm L-glutamine at 37 °C with 5% CO_2_. The human primary fibroblast cells were maintained in DMEM (Sigma-Aldrich, 1000 mg/L glucose, D5546) containing 20% FBS, 1% penicillin/streptomycin, 2 mm L-glutamine, and 10 mm HEPES at 37 °C with 5% CO_2_.

MCF7, SKBR-3, and 4T1 cells were purchased from the American Type Culture Collection (ATCC). Cells were regularly checked for mycoplasma contamination. In the cellular experiments, control cells received vehicle (0.001% DMSO in medium) but no LCA. All cellular experiments were performed in the presence of 10% FBS unless stated otherwise.

### 2.3. Proliferation Assay

Cellular proliferation was assessed using a sulforhodamine assay, as described in Reference [42]. Cells were seeded in a 96-well plates (4T1—1500 cells/well) and treated with the primary bile acids, CA and CDCA (0.01–10 µm) and NRF2 activator, RA839 (5 µm and 10 µm), or GSH and NAC antioxidants (5 mm) in the presence of LCA (0.3 µm) for 2 days. The cells were then fixed by the addition of trichloroacetic acid at a final concentration of 10% and were incubated for 1 h at 4 °C. Cells were washed with water and stained with 0.4% (*w*/*v*) sulphorhodamine B solution in 1% acetic acid. Unbound dye was removed by washing with 1% acetic acid. Bound stain was solubilized with 10 mm Tris base and the absorbance was measured at 540 nm.

### 2.4. Real-Time Quantitative PCR (RT-qPCR)

RNA isolation and RT-qPCR reactions were performed similarly to in Reference [43]. Total RNA was isolated from cells and tumor samples using TRIzol reagent (Invitrogen Corporation, Carlsbad, CA, USA). RNA (2 µg) was reverse transcribed using a High Capacity cDNA Reverse Transcription Kit (Applied Biosystems, Foster City, CA, USA) according to the manufacturer’s instructions. The qPCR was performed with qPCRBIO SyGreen Lo-ROX Supermix (PCR Biosystems Ltd., London, UK) on a Light-Cycler 480 Detection System (Roche Applied Science, Basel, Switzerland). The 36B4 gene was used for normalization. Primers are listed in Table 1.

### 2.5. SDS-PAGE and Western Blotting

Protein isolation, SDS-PAGE, and western blotting were performed similarly to in Reference [44]. Cells were lysed in RIPA buffer (50 mm Tris, 150 mm NaCl, 0.1% SDS, 1% TritonX 100, 0.5% sodium deoxycolate, 1 mm EDTA, 1 mm Na_3_VO_4_, 1 mm NaF, 1 mm PMSF, protease inhibitor cocktail). Protein samples (30–50 µg) were separated on 10% SDS polyacrylamide gels and electrotransferred onto nitrocellulose membranes. After blocking for 1 h with TBST containing 5% BSA, the membranes were incubated with primary antibodies overnight at 4 °C. After washing with 1× TBST solution, the membranes were probed with IgG HRP-conjugated secondary antibodies (Cell Signaling Technology, Inc. Beverly, MA, USA 1:2000). Bands were visualized by enhanced chemiluminescence reaction (SuperSignal West Pico Solutions, Thermo Fisher Scientific Inc., Rockford, IL, USA). Densitometry was performed using the Image J software [45]. Antibodies used in this study are listed in Table 2.

**Figure 1:**
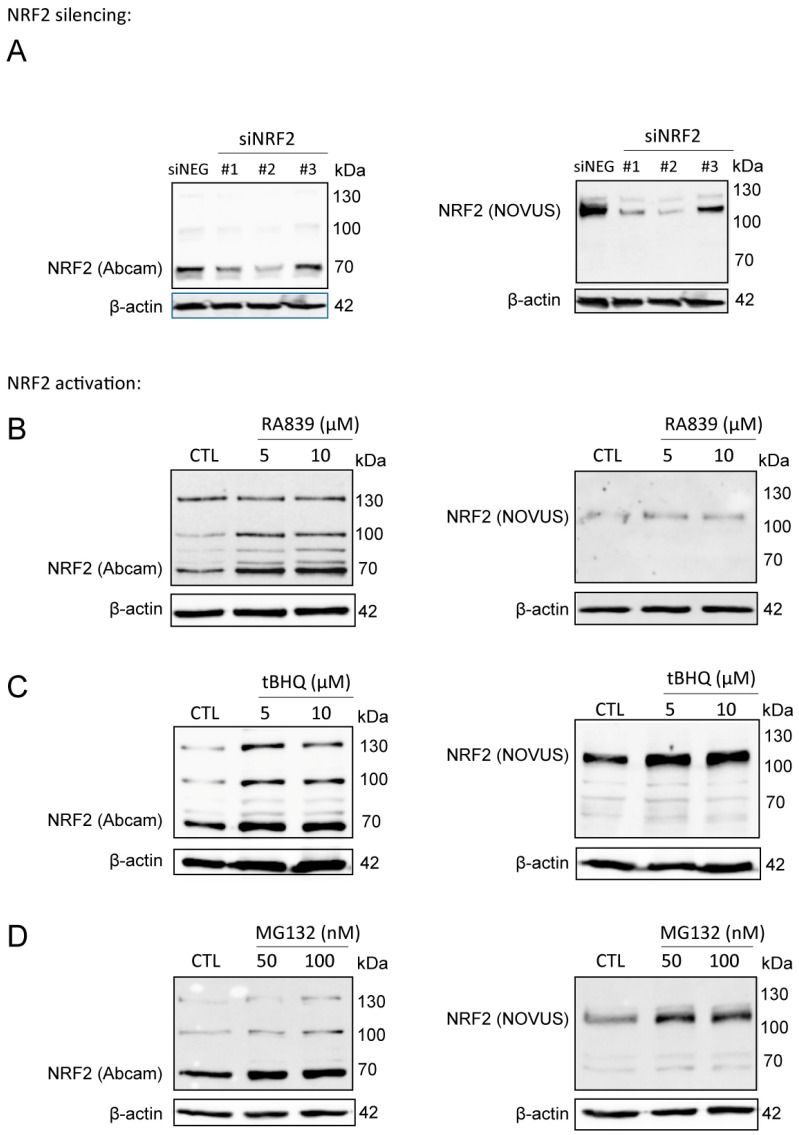
NRF2 antibody validation. (**A**) NRF2 expression was silenced in MCF7 cells by transiently transfecting NRF2-specific siRNAs or a negative control siRNA for 48 h, then NRF2 protein expression was determined using two different antibodies (Abcam: ab31163; NOVUS: NBP1-32822). (**B**–**D**) 4T1 cells were treated with NRF2 activators, RA839 or tBHQ, or MG-132, a proteasome inhibitor, in the concentrations indicated for 48 h, then NRF2 protein expression was determined by western blotting using two different antibodies (Abcam: ab31163; NOVUS: NBP1-32822).

We used the Abcam (ab31163) antibody in our studies, which we validated, due to the ambiguity in the molecular weight of NRF2. The calculated molecular weight of NRF2 is 68 kDa, however, recent studies have claimed that NRF2 has a molecular weight of ~130 kDa [46,47]. In the validation studies, we used a second NRF2 antibody (Novus, NBP1-32822) which, according to the literature [46,47], recognizes the ~130 kDa form of NRF2. The signal from both antibodies decreased when NRF2 was silenced by siRNA (Figure 1A). NRF2 protein was stabilized when NRF2 activators (RA839 or tBHQ) (Figure 1B,C) or a proteasome inhibitor (MG132) (Figure 1D) were administered to the cells. Both high and low molecular weight bands behaved in a similar fashion regardless of the antibody used. In the upcoming experiments, we used the Abcam ab31163 antibody and quantitation was done based on the ~70 kDa band.

### 2.6. Determination of Lipid Peroxidation

Lipid peroxidation was assessed using the thiobarbituric acid-reactive substances (TBARS) assay as described in [48]. The 4T1 cells were seeded in T150 flasks and were treated with LCA (0.3 µm) or NRF2 activator (5 µm and 10 µm) together with LCA (0.3 µm) for 2 days. Cells were washed with PBS, scraped, and collected by centrifugation. After adding 8.1% SDS, 20% acetic acid, 0.8% thiobarbituric acid (TBA), and distilled water to the cell pellet, the sample was incubated at 96 °C for 1 h. Samples were cooled down and centrifuged, and then the absorbance of the supernatant was measured at 540 nm. As a marker of lipid peroxidation, levels of 4-hydroxynonenal (4HNE)-modified proteins were also determined using western blotting.

### 2.7. Transfections

Cells were seeded in 24-well plates (MCF7—50.000 cell/well). On the following day, cells were transiently transfected with TGR5, CAR, VDR, or PXR siRNA or the negative control at a final concentration of 30 nM using Lipofectamine RNAiMAX transfection reagent (Invitrogen). Cells were incubated with transfection complexes in medium containing LCA (0.3 μm) for 48 h. CTL stands for vehicle-treated (0.001% DMSO in medium), non-transfected cells, while NEG stands for negative control siRNA-transfected, LCA-treated cells.

### 2.8. ABTS Decoloration Assay

Preparation of 2,2′-Azino-bis-3-ethylbenzothiazoline-6-sulfonic acid (ABTS) was performed as described [49]. The absorbance of ABTS solution was adjusted to 1.2. LCA and ascorbic acid were dissolved and diluted in DMSO. Five microliters of samples were added to the wells of 96-well half area microplates, then 50 μL of ABTS solution was added to the wells. Samples were incubated at RT for 30 min. Absorbance was measured with Tecan Spark multi-label reader (405 nm). Antioxidant activity was expressed as a percentage of control (DMSO-treated) samples. The 9% DMSO in ATBS buffer was used for dilution at all concentrations. An ascorbic acid concentration series was used as a positive control.

### 2.9. Database Screening

The kmplot.com database [50] was used to study the link between gene expression levels (CAR, TGR5, NRF2, KEAP1, iNOS, nNOS, and NOX4) and breast cancer survival in humans. Probe numbers are indicated in the corresponding tables. The GEO database of the NCBI was assessed using the following keywords: CAR + breast cancer, iNOS + breast cancer, nNOS + breast cancer, NRF2 + breast cancer, and TGR5 + breast cancer.

### 2.10. Tissue Microarray, Immunohistochemistry, and Analysis

This study was authorized by the institutional ethical body. Tissue microarray (TMA) and immunohistochemistry were performed as described in Reference [51]. The TMA was built from the archived tissue blocks of 88 breast cancer patients. We took three replicate samples from each block and we evaluated the staining using the H-score system [52]. For immunohistochemistry, the protocol of *Leica Bond Max™* was used. The antibodies and the conditions used are summarized in Table 3.

### 2.11. Animal Study

Animal experiments were authorized by the local and national ethical board (reg. 1/2015/DEMÁB) and were performed to conform to the relevant EU and US guidelines. We re-analyzed samples from a previous experiment in line with the 3R principles.

We assessed the effects of the supplementation of LCA on tumor growth and behavior by grafting 4T1 cells to Balb/c female mice, as described in Reference [19]. LCA was administered by oral gavage, in a dose of 15 nmol once a day. This dose corresponds to the serum reference concentration of LCA [19]. After two weeks, the mice were sacrificed by cervical dislocation, and tumor and metastases were harvested for subsequent analysis.

The 4T1 cells were suspended (2 × 10^6^/mL) in ice-cold PBS–matrigel (1:1, Sigma-Aldrich) at a 1:1 ratio. From this suspension, female BALB/c mice received 50 µL injections to their second inguinal fat pads on both sides (10^5^ cells/injection). Tumor growth and animal wellbeing were monitored daily.

Animals received daily oral LCA treatments. LCA stock was prepared in 96% ethanol at 100× concentration (7.5 mm) for storage at −20 °C. LCA stock was diluted each day to a working concentration of 75 µm in sterile PBS immediately before the treatment. Ethanol vehicle (1% in PBS) was prepared and diluted similarly. Animals received a daily oral dose of 200 µL/30 g bodyweight from the LCA solution or the vehicle. Researchers administered LCA and vehicle solutions blind. Treatment was administered every day at the same time between 8 a.m. and 10 a.m.

Experimental animals were female BALB/c animals between 8–10 weeks of age (20–25 g). Mice were randomized for all experiments. Animals were bred in the “specific pathogen free” zone of the Animal Facility at the University of Debrecen, and kept in the “minimal disease” zone during the experiment. Animal studies have been reported in compliance with the ARRIVE guidelines [53,54].

Mice were purchased from Jackson Laboratories (Bor Harbor, ME, USA) and were subsequently bred at the animal facility of the University of Debrecen. No more than six mice were housed in each cage (standard block shape 365 × 207 × 140 mm, surface 530 cm^2^; 1284 L Eurostandard Type II. L from Techniplast) with Lignocel Select Fine (J. Rettenmaier und Söhne, Germany) as bedding. Mice had paper tubes to enrich their environment. The dark/light cycle was 12 h, and temperature 22 ± 1 °C. Cages were changed once a week, on the same day. Mice had ad libitum access to food and water (sterilized tap water). The animal facility was overseen by a veterinarian. A total of 28 mice were used in this study and group sizes are indicated in the figure captions.

### 2.12. Statistical Analysis

We used a two-tailed Student’s *t*-test for the comparison of two groups unless stated otherwise. Fold data were log_2_ transformed to achieve normal distribution. For multiple comparisons, one-way analysis of variance test (ANOVA) was used followed by Tukey’s or Dunnett’s honestly significant (HSD) post hoc test. Data are presented as mean ± SEM unless stated otherwise. Statistical analysis was done using GraphPad Prism VI software. Correlation studies were done using Pearson correlation test and linear regression. Mitotic index was log_2_ transformed before the analysis. Values of *p* < 0.05 were considered statistically significant. Calculations were performed by R project [55] version 3.5.2.

## 3. Results

### 3.1. Lithocholic Acid Inhibited NRF2 Activation

**Figure 2:**
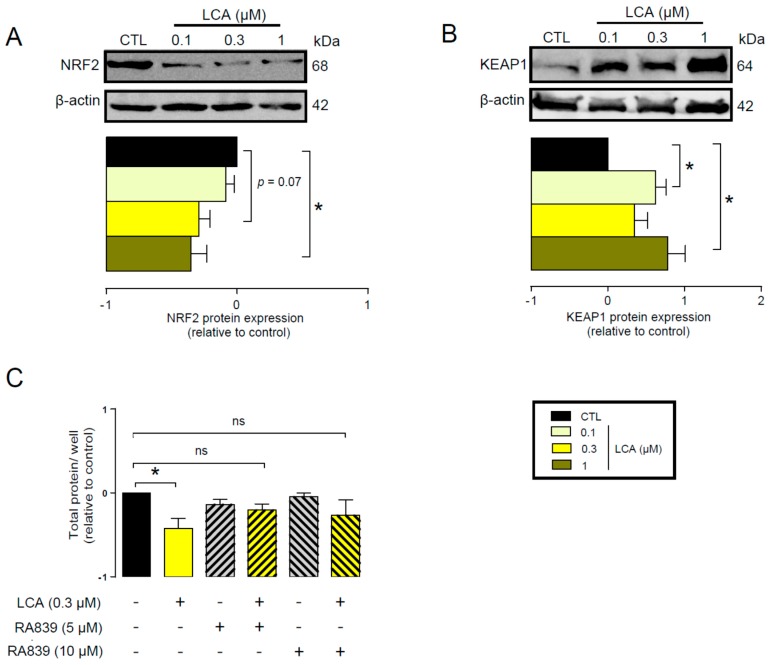
LCA inhibited the NRF2/KEAP1 system. (**A**,**B**) The 4T1 cells were treated with LCA in the concentrations indicated for 48 h, then (**A**) NRF2 and (**B**) KEAP1 proteins were analyzed by western blotting. (*n* = 3, upper panel: representative figure, lower panel: densitometric analysis of western blots from independent experiments). (**C**) The 4T1 cells were treated with 0.3 μm LCA and/or the NRF2 activator, RA839, in the concentrations indicated for 48 h, then total protein concentration was determined by sulforhodamine B assay (*n* = 5). Data are plotted as mean ± SEM. * indicates *p* < 0.05, control vs. LCA-treated groups. (ns, not significant; KEAP1, Kelch-like ECH associating protein 1; LCA, lithocholic acid; NRF2, nuclear factor).

**Figure 3:**
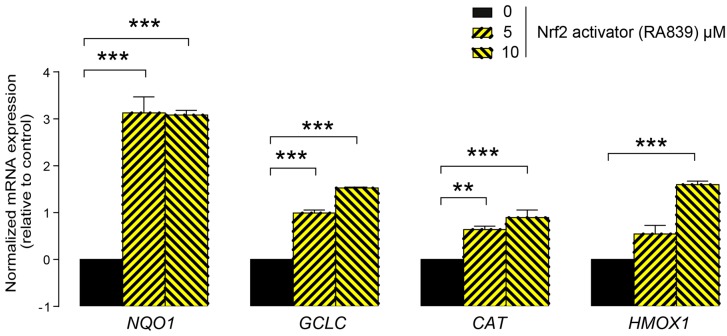
Pharmacological activation of NRF2 induced the expression of NRF2 target genes. The 4T1 cells were treated with the NRF2 activator, RA839, in the concentrations indicated for 48 h, then the expressions of NRF2 target genes, *NQO1*, *GCLC*, *CAT*, and *HMOX1,* were determined using RT-qPCR (*n* = 3). Abbreviations: NAD(P)H quinone dehydrogenase 1 (*NQO1*), glutamate–cysteine ligase catalytic subunit (*GCLC*), catalase (*CAT*), and heme oxygenase 1 (*HMOX1*). Data are plotted as mean ± SD. ** and *** indicate statistically significant differences between control and RA839-treated groups at *p* < 0.01 or *p* < 0.001, respectively.

First, we assessed whether LCA administration could influence the expression of key elements in the antioxidant NRF2/KEAP1 pathway. The LCA concentrations used in the experiments corresponded to the normal concentrations of LCA in human breast tissue (0.1–1 μm) [41]. LCA treatment of 4T1 mouse breast cancer cells decreased NRF2 protein levels (Figure 2A) while upregulating protein expression of the NRF2 repressor, KEAP1 (Figure 2B). The pharmacological activation of NRF2 by RA839 abolished the anti-proliferative effects of LCA (Figure 2C). We assessed the effectiveness of RA839 by measuring the mRNA expression of a set of NRF2-regulated genes: NAD(P)H quinone dehydrogenase 1 (*NQO1*), glutamate–cysteine ligase catalytic subunit (*GCLC*), catalase (*CAT*), and heme oxygenase 1 (*HMOX1*), (Figure 3). Taken together, these results show that decreased NRF2 expression played a key role in eliciting the cytostatic effects of LCA.

### 3.2. LCA Treatment Induced Oxidative Stress by Reducing NRF2 Expression

**Figure 4:**
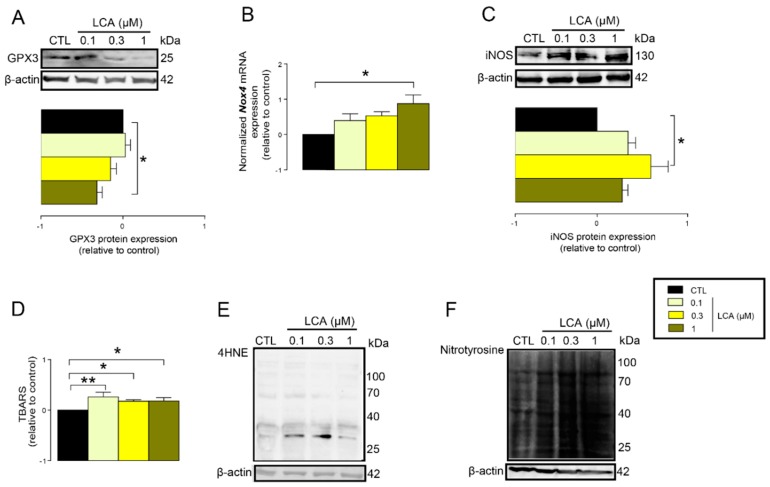
LCA-induced oxidative stress in 4T1 breast cancer cells. (**A**–**F**) The 4T1 cells were treated with LCA for 48 h, then the indicated measurements were performed. (**A**) GPX3 antioxidant protein expression was analyzed by western blotting (*n* = 4). (**B**) *NOX4* mRNA expression was determined in RT-qPCR (*n* = 4). (**C**) The level of iNOS protein was detected by western blotting (*n* = 3). (**D**) Lipid peroxidation was measured by determining TBARS (*n* = 3). (**E**) The 4HNE levels were determined by western blotting (representative figure, *n* = 3). (**F**) Nitrotyrosine was detected in western blotting (*n* = 3). In the cases of 4HNE and nitrotyrosine, similar results were obtained in three independent experiments. Data are plotted as mean ± SEM. * and ** indicate *p* < 0.05 or *p* < 0.01, control vs. LCA-treated groups. (GPX3, glutathione peroxidase 3; LCA, lithocholic acid; NOX4, NADPH oxidase 4; TBARS, thiobarbituric acid reactive substances; 4HNE, 4-hydroxynonenal).

The previous results suggested that LCA treatment may impair cellular antioxidant defenses. In line with that, we found that the protein expression of glutathione peroxidase-3 (GPX3), a key antioxidant protein, decreased upon LCA treatment (Figure 4A). Oxidative stress is an imbalance between antioxidant and pro-oxidant genes. Thus, we assessed whether LCA can induce expression of pro-oxidant genes. LCA induced the mRNA expression of NADPH oxidase 4 (*NOX4*), a major ROS producing enzyme (Figure 4B), and inducible NO synthase (iNOS), a major source of nitric oxide in cells (Figure 4C).

**Figure 5:**
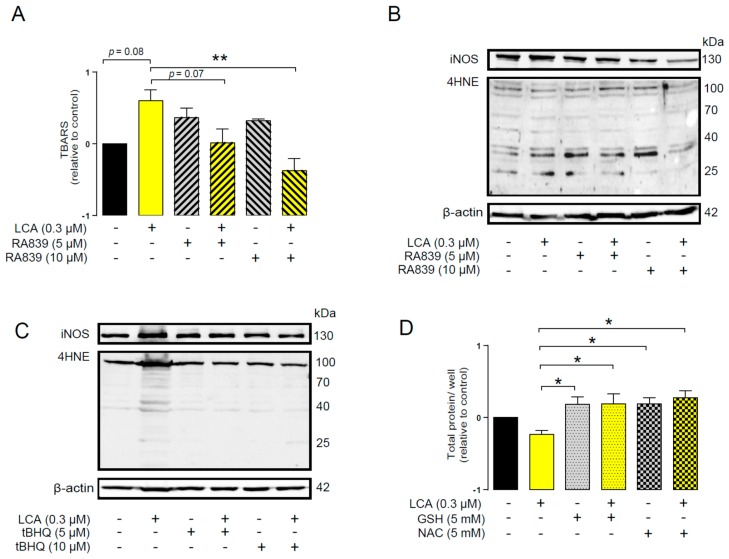
NRF2 activation modulated LCA-induced oxidative stress responses in 4T1 breast cancer cells. The 4T1 cells were treated with 0.3 μm LCA and the NRF2 activator RA839 or tBHQ in the concentrations indicated for 48 h. Lipid peroxidation was determined by measuring (**A**) TBARS (*n* = 4) and (**B**,**C**) 4-HNE levels using western blotting (*n* = 3). (**D**) The 4T1 cells were treated with LCA (0.3 μm) and/or GSH and NAC (both at 5 mm) antioxidants for 48 h, then total protein concentration was determined using the sulforhodamine B assay (*n* = 3). For statistical analysis ANOVA test was used followed by the Dunnett post-hoc test, where all groups were compared to the LCA-treated cohort. Data are plotted as mean ± SEM. ** *p* < 0.01, LCA vs. LCA/NRF2-activator-treated groups (GSH, reduced glutathione; LCA, lithocholic acid; NAC, N-acetylcysteine; TBARS, thiobarbituric acid reactive substances; 4HNE, 4-hydroxynonenal).

**Figure 6:**
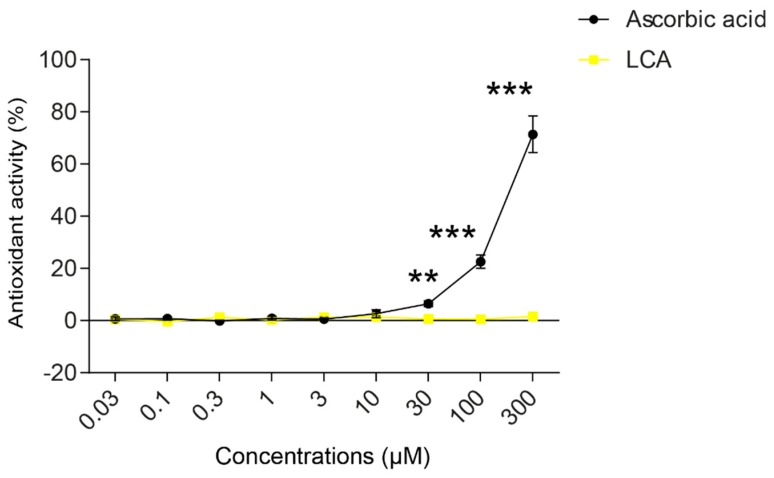
LCA did not act as an antioxidant. The ABTS radical scavenging assay was done in 96-well plates using triplicate samples. LCA was tested in a concentration range of 0.03–300 μm. Ascorbic acid was used as a positive control. Antioxidant activity was expressed as the percentage of control samples. Means of three independent experiments ± SD are presented. ** and *** indicate a statistically significant difference between control and ascorbic acid-treated groups at *p* < 0.01 or *p* < 0.001, respectively.

An imbalance between pro-oxidant and antioxidant systems leads to oxidative or nitrosative stress. We detected increased lipid and protein oxidation after LCA treatment, as shown by increases in thiobarbituric acid reactive species (TBARS) (Figure 4D) and 4-hydroxynonenal adducts (4HNE) (Figure 4E) [56]. Moreover, increased expression of iNOS suggested increased production of ONOO^−^ [57,58] and the subsequent enhancement of nitrosative stress. Nitrotyrosine levels were increased in LCA-treated cells (Figure 4F). Importantly, the activation of NRF2 by RA839 or tBHQ prevented both increases in TBARS (Figure 5A) and 4HNE (Figure 5B,C) and decreases in iNOS expression (Figure 5B,C) when applied in combination with LCA. Finally, the thiol antioxidants glutathione (GSH) and N-acetyl-cysteine (NAC) blunted the LCA-elicited anti-proliferative effects (Figure 5D) but did not impact other LCA-mediated cancer hallmarks, such as cancer cell metabolism or epithelial-to-mesenchymal transition [19]. LCA had no direct antioxidant effects in the concentrations we used in the current study (0.1–1 µm), or at higher concentrations (up to 300 µm), where ascorbic acid readily acted as an antioxidant (Figure 6).

**Figure 7:**
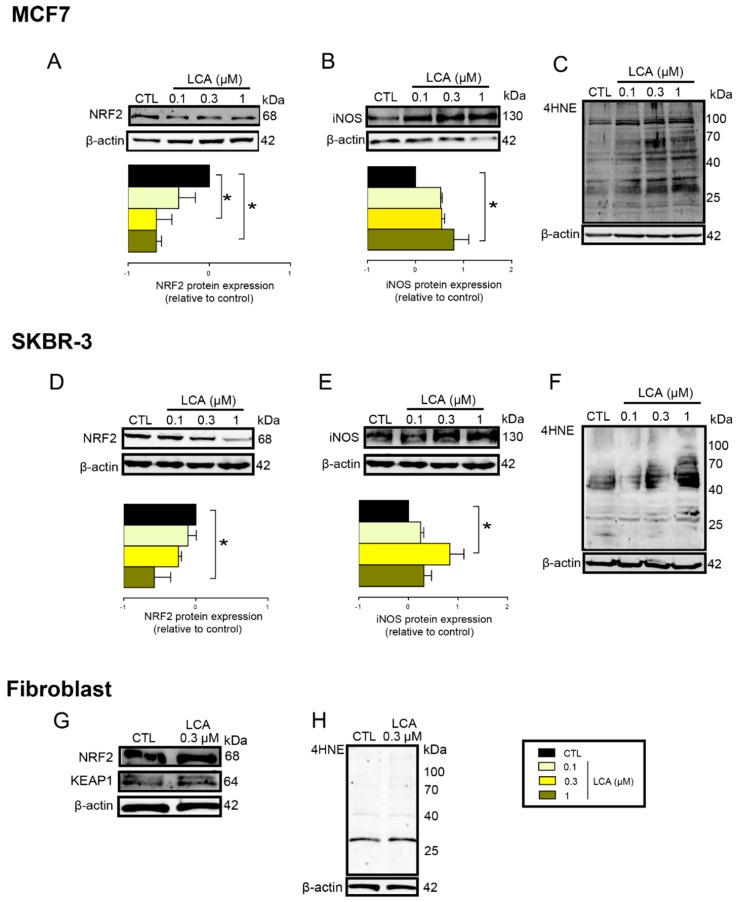
LCA induced oxidative stress in MCF7 and SKBR-3 human breast cancer cell lines, but not in primary fibroblasts. (**A**–**C**) The MCF7 cells were treated with LCA in the concentrations indicated for 48 h, then (**A**) NRF2, (**B**) iNOS protein expression, and (**C**) 4HNE were determined by western blotting (*n* = 3). (**D**–**F**) The SKBR3 cells were treated with LCA for 48 h, then (**D**) NRF2, (**E**) iNOS, and (b) 4HNE expressions were determined by western blotting (*n* = 3). Upper panels: representative figures. Lower panels: densitometric analysis of western blots. In the case of 4HNE, similar data were obtained in three independent experiments. (**G**,**H**) Fibroblast cells were treated with 0.3 µm LCA for 48 h then (**G**) NRF2, KEAP1, and (**H**) 4HNE expressions were determined by western blotting. Data are plotted as mean ± SEM. * *p* < 0.05, control vs. LCA-treated. (LCA, lithocholic acid; NRF2, nuclear factor, erythroid 2-like 2; 4HNE, 4-hydroxynonenal; iNOS, inducible nitric oxide synthase).

**Figure 8:**
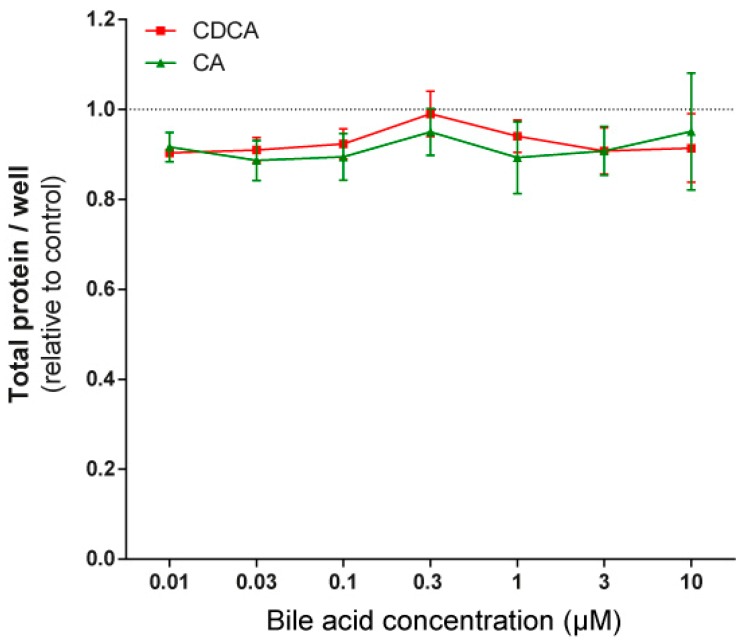
Primary bile acids did not affect the proliferation of 4T1 breast cancer cells. The 4T1 cells were treated with CA and CDCA in the concentrations indicated for 48 h, then total protein concentrations were determined by sulforhodamine B assay (*n* = 4). Data are plotted as mean ± SEM. (CA, cholic acid; CDCA, chenodeoxycholic acid).

To assess whether the phenomena described above were restricted to 4T1 cells, the effects of LCA were assayed in MCF7 and SKBR3 cancer cell lines. LCA treatment decreased NRF2 expression (Figure 7A,D) and increased iNOS expression (Figure 7B,E) and 4HNE signals (Figure 7C,F) in both cell lines, similarly to our observations in 4T1 cells. LCA did not modulate the expression of NRF2, KEAP1, or 4HNE in primary, non-transformed human fibroblasts (Figure 7G,H). Primary bile acids, in concentrations corresponding to their serum reference concentrations, did not significantly reduce cancer cell proliferation (Figure 8).

### 3.3. LCA-Elicited Oxidative Stress Was Mediated by TGR5 and Partially by CAR Receptor

**Figure 9:**
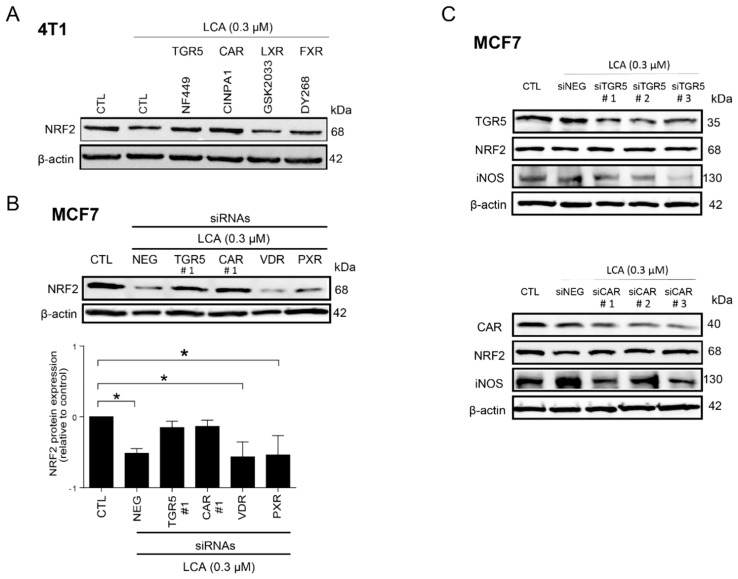
LCA-induced oxidative stress responses were mediated by TGR5 and by CAR receptors. (**A**) The 4T1 cells were treated with 0.3 μm LCA and NF449, CINPA1, DY268, or GSK2033 at a final concentration of 5 μm for 48 h, then NRF2 protein expression was detected using western blotting (representative figure, *n* = 2). (**B**,**C**) TGR5, CAR, VDR, and PXR bile acid receptors were silenced in MCF7 cells by transiently transfecting the cells with the corresponding siRNA or a negative control siRNA. After 48 h, protein expressions of (**B**,**C**) NRF2 and (**C**) iNOS were determined by western blotting (*n* = 3). Data are plotted as mean ± SEM. * *p* < 0.05, control vs. LCA/siRNA treated. (CAR, constitutive androstane receptor; FXR, farnesoid X-activated receptor; LCA, lithocholic acid; LXR, liver X nuclear receptor; NRF2, nuclear factor, erythroid 2-like 2; TGR5/GPBAR1, G protein-coupled bile acid receptor 1/Takeda G-protein coupled receptor; VDR, vitamin D receptor).

Next, we aimed to identify the receptors responsible for the effects of LCA. Several nuclear receptors and the Takeda G-protein coupled receptor (TGR5) can bind and respond to LCA [19]. First, we used pharmacological agents designed to inhibit LCA receptors, including CINPA1 to inhibit the constitutive androstane (CAR) receptor, DY268 to inhibit the farnesyl-X receptor (FXR), GSK2033 to inhibit the liver X receptor (LXR), and NF449, a G_sα_-selective antagonist that can inhibit the downstream signaling of the TGR5 receptor. LCA-mediated reduction in NRF2 protein expression was blocked by NF449 and CINPA1, while the other inhibitors (GSK2033 and DY268) were ineffective (Figure 9A).

The pharmacological experiments above were complemented by siRNA depletion experiments. Other possible LCA receptors, including the vitamin D receptor (VDR) and the pregnane X receptor (PXR), were also assessed. To provide a comprehensive view, we silenced TGR5, CAR, VDR, and PXR in MCF7 cells. Silencing of TGR5 and CAR efficiently blocked the LCA-induced decreases in NRF2 protein (Figure 9B,C), similarly to the pharmacological agents. Silencing of VDR and PXR receptor did not affect LCA-mediated reduction of NRF2 protein levels (Figure 9B). Next, we assessed iNOS protein level after silencing of TGR5 and CAR receptors. Silencing of either TGR5 or CAR receptors blunted the LCA-induced decrease in NRF2 and increased iNOS expression (Figure 9C). These data indicate that LCA exerts its effects through the TGR5 receptor and CAR receptor.

### 3.4. LCA Supplementation Suppressed Antioxidant Defense in an Animal Model of Breast Cancer

As a next step, we assessed whether the supplementation of LCA could hamper the redox status of tumors in an in vivo setting. We re-analyzed samples from a previous study we published [19]. In that study, we grafted Balb/c female mice with 4T1 cells and supplemented mice orally with 15 nmol LCA daily for two weeks. At the end of the study, mice were sacrificed and tumors were harvested. In the previous study [19], we showed that LCA supplementation in vivo inhibits tumor growth, metastasis formation, epithelial-to-mesenchymal transition, and bioenergetic changes [19]. These findings are supported by patient observation and wet chemistry experiments that have been carried out by others [59,60,61].

**Figure 10:**
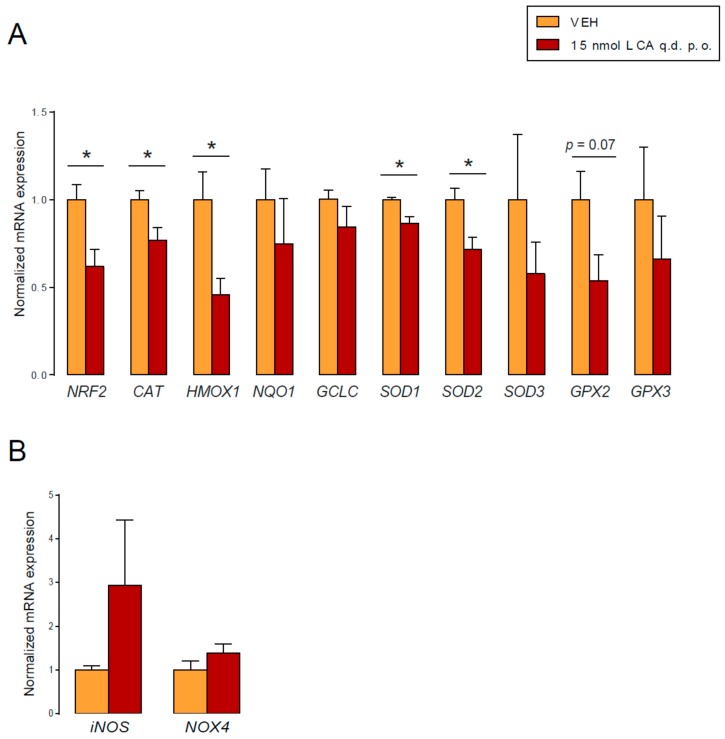
LCA modulated the expression level of antioxidant and pro-oxidant genes in vivo. (**A**,**B**) Female Balb/c mice were grafted with 4T1 cells and treated with LCA (15 nmol q.d. p.o.) or vehicle (VEH) (*n* = 5/5) for 18 days. The mRNA expression levels of the indicated genes were determined in tumors using RT-qPCR. Error is depicted as SEM. * indicates statistically significant differences between vehicle and treated groups at *p* < 0.05. (CAT, catalase; GCLC, glutamate–cysteine ligase catalytic subunit; GPX2, glutathione peroxidase 2; GPX3, glutathione peroxidase 3; HMOX1, heme oxygenase 1; iNOS, inducible NO synthase; NOX4, NADPH oxidase 4; NQO1, NAD(P)H quinone dehydrogenase 1; NRF2, nuclear factor, erythroid 2-like 2; SOD1, superoxide dismutase 1; SOD2, superoxide dismutase 2; SOD3, superoxide dismutase 3).

In the tumors of control and LCA-treated mice, we assessed the expression of anti- and pro-oxidant genes. LCA supplementation reduced the expression of NRF2 and a set of antioxidant genes: catalase (CAT), glutamate–cysteine ligase catalytic subunit (GCLC), glutathione peroxidase 2 (GPX2), glutathione peroxidase 3 (GPX3), heme oxygenase 1 (HMOX1), inducible NO synthase (iNOS), NADPH oxidase 4 (NOX4), NAD(P)H quinone dehydrogenase 1 (NQO1), nuclear factor, erythroid 2-like 2 (NRF2), superoxide dismutase 1 (SOD1), superoxide dismutase 2 (SOD2), and superoxide dismutase 3 (SOD3) (Figure 10A). Furthermore, we observed a non-significant increase in iNOS and NOX4 expression in the LCA-treated mice (Figure 10B). These results suggest that LCA can exert its activity on the redox balance of cancer cells in vivo, with beneficial effects for the outcome of the disease.

### 3.5. Elements of the LCA-Elicited Anticancer Pathway Correlated with Stage, Grade, and Receptor Status of the Disease

**Figure 11:**
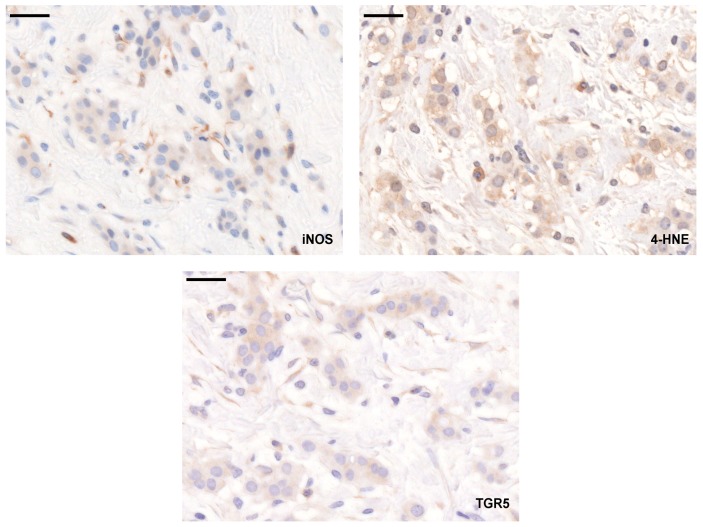
Staining pattern of the antibodies used in the study. Breast cancer specimens in a TMA were stained with the antibodies indicated, and the immune reactions were developed using DAB. The black line indicates 50 µm.

We assessed the expression of the LCA-elicited oxidative/nitrosative stress markers (TGR5, iNOS, and 4HNE) using a tissue microarray (TMA) made up of tumor samples from 88 breast cancer patients. In parallel, we assessed the available public expression databases, GEO Profiles (https://www.ncbi.nlm.nih.gov/geoprofiles/) and kmplot.com [50]. The typical staining pattern of the antibodies is shown in Figure 11.

**Figure 12:**
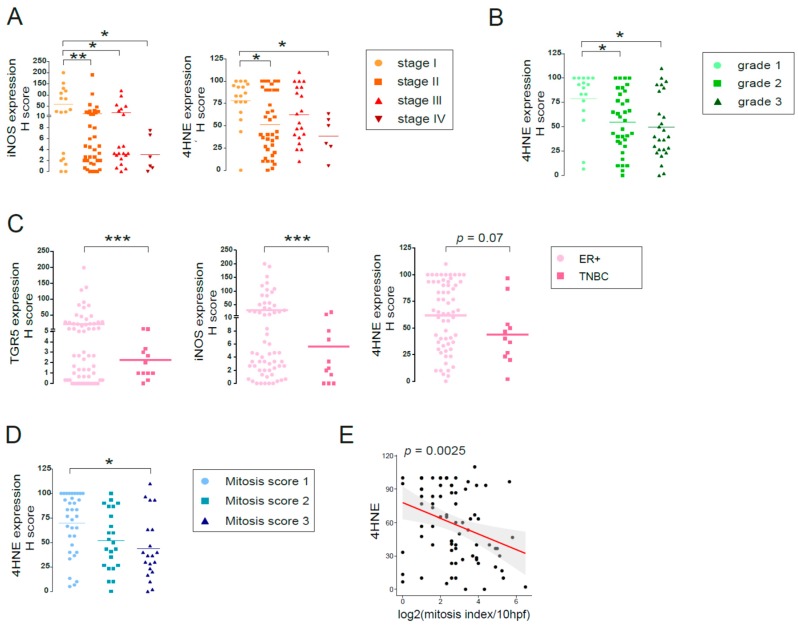
LCA-induced oxidative stress pathway is protective in human breast cancer. (**A**–**D**) The expression levels of oxidative/nitrosative stress markers were analyzed in tumor samples of 88 breast cancer patients using tissue microarray (TMA), as indicated on the graphs. (**A**) Patients were stratified based on disease stage (Stage I–IV), (**B**) pathological grade of the disease (Grade 1–3), (**C**) ER+ vs. TNBC cases, and (**D**) mitosis score (mitosis score 1–3), and the expression level of indicated markers were determined using IHC analysis. (**E**) Linear regression analyses for the correlation between 4HNE expression levels and the mitotic index were determined using R project. TGR5: Pearson r = −0.13; *p* = 0.26; iNOS: Pearson r = −0.16; *p* = 0.16; 4HNE: Pearson r = −0.34; *p* = 0.0025. Line represents linear regression of data (TGR5: y = 25 − 3x; iNOS: y = 38 − 4.7x; 4HNE: y = 78 − 7x). *, **, and *** indicate *p* < 0.05, *p* < 0.01 or *p* < 0.001, differences between patient groups. (TGR5/GPBAR1, G protein-coupled bile acid receptor 1/Takeda G-protein coupled receptor; 4HNE, 4-hydroxynonenal).

First, we stratified patients for the TMA based on disease stage from stage I to stage IV, based on the primary tumor size, the lymph node involvement, and distant metastasis (as in Reference [27]). In our previous study [19], we showed that LCA production by the gut microbiome decreased in breast cancer and that the capability of the microbiome to synthesize LCA correlated with the disease stage. Levels of iNOS and 4HNE decreased in stage II and stage III patients compared to stage I patients and further decreased in stage IV patients (Figure 12A).

Next, we stratified patients based on the pathological grade (Nottingham grade) of the disease. 4HNE expression significantly decreased in grade II and grade III patients compared to grade I patients (Figure 12B). In line with that, high expression of KEAP1 was associated with better survival for grade II patients, and high CAR expression was associated with better survival for grade III patients (Table 4).

**Figure 13:**
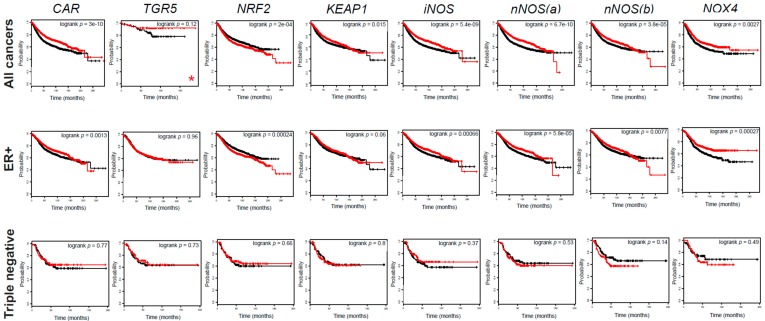
The components of the LCA–TGR/CAR pathway are protective in human breast cancer. The impact of *CAR*, *TGR5*, *NRF2*, *KEAP1*, *iNOS*, *nNOS*, and *NOX4* expression on survival in breast cancer patients was evaluated by assessing the kmplot.com database. All cancers, ER+, and triple negative cases were analyzed. The ER+ population also included those patients where ER status was derived from gene expression data. Triple negative cases included patients with ER− status (including those patients where ER status was derived from gene expression data), PR− status, and HER2− status. The red line indicates high expression while the black line indicates low expression. Probe IDs, numbers at risk, and hazard ratios are collected in Table 5. *nNOS*(a) represents 207309_at probe id, while *nNOS*(b) represents 207310_at probe set. * indicates that grade 1 patients are depicted. (CAR, constitutive androstane receptor; ER, estrogen receptor; KEAP1, Kelch-like ECH associating protein 1; NOX4, NADPH oxidase 4; NRF2, nuclear factor, erythroid 2-like 2; TGR5/GPBAR1, G protein-coupled bile acid receptor 1/Takeda G-protein coupled receptor; iNOS, inducible nitric oxide synthase; nNOS, neuronal nitric oxide synthase).

We also stratified patients as triple negative (TNBC; ER− PR− HER2−) or ER+ cases. The expression of TGR5, iNOS, and 4HNE decreased in TNBC cases as compared to ER+ cases (Figure 12C). In line with that, higher expression of *CAR*, *KEAP1*, *iNOS*, *nNOS,* and *NOX4* or lower expression of *NRF2* was associated with better survival when we assessed all patients or ER+ positive cases, but not TNBC cases (Figure 13, Table 4 and Table 5).

Finally, we grouped patients as a function of the mitosis score. Staining for 4HNE, the most direct indicator of tissue oxidative stress, decreased as mitosis score increased (Figure 12D). Furthermore, 4HNE staining showed a strong negative correlation with the mitosis index (Figure 12E).

Taken together, LCA-elicited oxidative stress correlated well with the mitotic rate in breast cancer. Furthermore, the LCA-elicited cytostatic system was hampered at higher breast cancer stages, higher grade carcinomas, or in TNBC cases that had a poor prognosis. In line with that, we identified cases in the TMA study where the staining for iNOS was well-maintained in the surrounding healthy breast tissue, but was lost in the cancerous tissues, or when NRF2 expression was upregulated in the cancerous tissue as compared to the neighboring healthy tissue. Further supporting our observations, we found a dataset in the GEO database [62] in which the expression of TGR5 receptor was shown to be lower in ductal in situ (DCIS) cases compared to control, healthy breast tissue.

## 4. Discussion

In breast cancer, the diversity of the gut microbiome is reduced [4]. The gut microbiome produces a large set of metabolites, and a subset of these metabolites (e.g., LCA or cadaverine) have anticancer effects on breast cancer cells [18,19,21,22,25,26]. In addition to the reduction in microbial diversity, the production of these antiproliferative metabolites is decreased in breast cancer patients [18,19]. Cadaverine and LCA exert their effects by modulating a plethora of cancer hallmarks. LCA reverts EMT, modulates cancer cell metabolism, induces anticancer immunity, and inhibits proliferation [19]. In supraphysiological concentrations, LCA induces cell death [21,22]. Interestingly, cadaverine, another cytostatic bacterial metabolite, does not induce oxidative stress [18].

We widened the scope of LCA-mediated effects by showing that, when LCA was applied in concentrations corresponding to tissue LCA concentrations [41], oxidative stress was increased through the downregulation of NRF2 and increased expression of pro-oxidative enzymes. In other words, LCA-induced increases in oxidative and nitrosative stress stem from an imbalance between pro-oxidant and antioxidant systems. Enhanced production of reactive species damages proteins and lipids. The production of reactive species has a primary role in slowing down breast cancer cell proliferation; however, in our study, antioxidants did not modulate LCA-induced epithelial-to-mesenchymal transition or cellular metabolism, which have been reported to be mediated by changes in oxidative stress in breast cancer [63]. A possible explanation for this discrepancy is the relatively small increase in reactive species in our models compared to the previous study [63]. LCA most likely mediates other pathways through NRF2, including the hypoxic response through hypoxia-inducing factors [63], mTORC1 [64], and proteostasis [65]. An interesting study showed that increased free radical production reprograms breast cancer cells from cancer stem cells to tumor stroma cells [66], suggesting that LCA could also induce a phenotypic switch to stroma cells.

The role of oxidative stress in breast cancer is controversial [67], as increases in reactive species can be pro-carcinogenic [68,69,70,71,72] as well as anti-carcinogenic [66,73,74] in breast cancer. In our study, higher expression of pro-oxidant genes and oxidative stress markers was associated with clinically benign forms of breast cancer (non-TNBC, low stage or low grade forms); however, pro-oxidant genes and oxidative stress markers gradually decreased in late stages, higher grades, or triple negative cases. In other words, the loss of the LCA–TGR5–oxidative stress pathway correlated with worse clinical outcomes. In accordance with that, higher CAR expression correlated with better survival. However, this benefit was lost in triple negative cases. Our results support the beneficial, cytostatic effects of oxidative stress. Our study correlates well with a metadata analysis study [33] which demonstrated that while increased oxidative stress, due to DNA damage and the consequent accumulation of mutations, represents a risk factor for breast cancer initiation, increased lipid peroxidation is associated with longer survival.

The NRF2–KEAP1 system and reactive species were shown to modulate the clinical behavior of breast cancer. NRF2 overexpression is an independent adverse prognostic factor for cancer recurrence and disease-free survival for breast cancer patients [75]. Moreover, there are SNPs in NRF2 and KEAP1 that modulate protein expression, and the polymorphisms associated with higher NRF2 or lower KEAP1 expression are associated with worse clinical outcomes [76,77,78]. In addition, oxidative stress and low NRF2 expression have been shown to potentiate the effects of doclitaxel [73] and other chemotherapeutic agents [79,80]. Apparently, the LCA-mediated pro-oxidative phenotype has a central role in mediating the clinical behavior of breast cancer. These findings correlate well with our observations from immunohistochemical and survival analysis. As an extension to the published studies, we demonstrated that TGR5, the receptor of LCA, is also crucial in mediating the effects of NRF2.

Our dataset revealed that estrogen and HER2 signaling affect the activity of the LCA-elicited pathways. The expression of the pro-oxidative iNOS and the oxidative stress marker, 4HNE, was lower in TNBC cases than in ER^+^ HER^+^ or HER^+^ cases. Similar trends were true for trace amino acid receptors (TAAR) 1, 2, 3, 5, 8, and 9, which are receptors of another bacterial cytostatic metabolite, cadaverine [18]. Although the molecular mechanism for the enhanced effectiveness of LCA in ER^+^/HER^+^ cancers has not been elucidated, other studies have substantiated the importance of the HER2 signaling pathway, such as in the finding that HER2 signaling induces iNOS and reduces cell proliferation [81].

The gut microbiome loses its diversity in breast cancer [4], and bacterial production and serum levels of cytostatic metabolites, such as LCA and cadaverine, decrease [18,19]. Low serum LCA content correlates with higher cancer cell proliferation rate [24]. A causative relationship between microbiome dysbiosis and breast cancer has been evidenced by a large set of studies [82,83,84,85,86,87] showing that antibiotic treatment increases breast cancer incidence. This observation has been further strengthened by our observation that primary bile acids were less effective in inducing cytostasis in breast cancer than LCA, a secondary bile acid. Therefore, reduction in microbiome biomass reduces LCA production, taking out an effective natural cytostatic compound.

Previously, we showed that LCA exerts its effects through the TGR5 receptor [19]. TGR5 overexpression has been shown to be beneficial in ampullary cancer by prolonging patient survival [88]. Our current data provide evidence that TGR5 expression and activation is a protective factor in breast cancer, and LCA is a physiological ligand for TGR5 in healthy individuals. Moreover, we identified CAR as an alternative LCA receptor. CAR overexpression in tumors is associated with better patient survival, similarly to TGR5 overexpression.

We have shown that LCA, a metabolite of the microbiome, induces oxidative and nitrosative stress by creating an imbalance in pro- and antioxidant systems in breast cancer cells. LCA and other similar bacterial metabolites (e.g., cadaverine) have properties that are similar to human hormones, as they are produced at a site (gut microbiome) different from the one(s) where they elicit their effects (breast). We have also provided evidence that the lower expression and activity of the LCA–TGR5 signaling system that characterizes triple negative breast cancers correlates with worse clinical outcomes in breast cancer. These findings have translational applicability by targeting TGR5/CAR signaling and oxidative stress for the treatment of breast cancer.

## 5. Conclusions

In this study, we showed that the cytostatic effects of the bacterial metabolite LCA depend on oxidative stress brought about by the downregulation of the NRF2/Keap1 system and the induction of iNOS, and, hence, nitrosative stress. LCA elicits these effects by activating TGR5 and CAR receptors. The LCA-induced oxidative stress pathway provides better survival in human breast cancer, and the downregulation of the expression of its components characterize the triple negative cases.

## Figures and Tables

**Table 1:** Primers used in the RT-qPCR reactions.

Gene Symbol	MURINE Forward Primer (5′–3′)	Murine Reverse Primer (5′–3′)
CAT	CCTTCAAGTTGGTTAATGCAGA	CAAGTTTTTGATGCCCTGGT
GCLC	GATTCGGGATGGGCAACT	AAAGGTATCTTGCCTCAGATATGC
GPX2	GTTCTCGGCTTCCCTTGC	TTCAGGATCTCCTCGTTCTGA
GPX3	GGCTTCCCTTCCAACCAA	CCCACCTGGTCGAACATACT
HMOX1	AGGCTAAGACCGCCTTCCT	TGTGTTCCTCTGTCAGCATCA
iNOS	GAAGTGCAAAGTCTCAGACATGG	GATTCTGGAACATTCTGTGCTGTC
NOX4	GCAGATTTACTCTGTGTGTTGCAT	TCCCATCTGTTTGACTGAGGT
NQO1	AGCGTTCGGTATTACGATCC	AGTACAATCAGGGCTCTTCTCG
NRF2	CATCAGGCCCAGTCCCTCAAT	CAGCGGTAGTATAÓCAGCCAGCT
SOD1	CCATCAGTATGGGGACAATACA	GGTCTCCAACATGCCTCTCT
SOD2	TGCTCTAATCAGGACCCATTG	GTAGTAAGCGTGCTCCCACAC
SOD3	CTCTTGGGAGAGCCTGACA	GCCAGTAGCAAGCCGTAGAA
36B4	AGATTCGGGATATGCTGTTGG	AAAGCCTGGAAGAAGGAGGTC

**Table 2:** Antibodies used in western blot analyses.

Antibody Symbol	Vendor	Dilution
NRF2	Abcam (ab31163)	1:1000
NRF2	Novus (NBP1-32822	1:1000
KEAP1	Cell Signaling (8047)	1:1000
GPX3	Abcam (ab104448)	1:1000
iNOS	Novus (NB300-605)	1:1000
4HNE	Abcam (ab46545)	1:1000
Nitrotyrosine	Millipore (06-284)	1:1000
TGR5/GPBAR1	Novus (NBP2-23669)	1:1000
CAR	Abcam (ab186869)	1:1000
ACTIN	Sigma-Aldrich (A3854)	1:20000

**Table 3:** Antibodies and conditions used in tissue microarray (TMA) analyses.

Antibody Symbol	Vendor	Antigen Retrieval	Dilution	Detection
NRF2	Abcam (ab31163)	Ventana BenchMark ULTRA/Roche Cell Conditioning 1 (CC1) 40 min, 95 °C	1:100	OptiView DAB IHC Detection kit/Roche
iNOS	ThermoFisher Scientific (PA5-16855)	Ventana BenchMark ULTRA/Roche Cell Conditioning 1 (CC1) 20 min, 95 °C	1:100	UltraView Universal DAB Detection kit/Roche
4HNE	Abcam (ab46545)	Ventana BenchMark ULTRA/Roche Cell Conditioning 1 (CC1) 20 min, 95 °C	1:1000	UltraView Universal DAB Detection kit/Roche
TGR5	GeneTEX (GTX100026, Hsinchu City, Taiwan)	pressure cooker (Avair) in 0.1 m citrate buffer at pH 6	1:1000	EnVision Flex (K8000, Dako, Santa Clara, CA, USA)

**Table 4:** Connection between iNOS, nNOS, TGR5, CAR, NRF2, and breast cancer patient survival. Bold numbers represent statistically significant results.

**CAR (207007_a_at)**	**HR (Hazard Ratio)**	***p*-Value (Log Rank Test)**
*All Breast Cancers, N = 3951*	0.7	**3.00 × 10^−10^**
*Grade 1, N = 345*	1.15	0.61
*Grade 2, N = 901*	0.88	0.3
*Grade 3, N = 903*	0.72	**0.0036**
*ER(+), N = 3082*	0.81	**0.0013**
*ER(−), N = 869*	0.62	**9.20 × 10^−6^**
*PR(+), N = 589*	0.72	0.063
*PR(−), N = 549*	0.9	0.49
*Lymph(+), N = 1133*	0.97	0.73
*Lymph(−), N = 2020*	0.99	0.93
*HER2(+), N = 252*	0.69	0.1
*HER2(−), N = 800*	1.13	0.37
*ER(+), PR(+), N = 577*	0.79	0.19
*ER(+), PR(+), Lymph node (+),N = 344*	0.99	0.97
*ER(+), PR(+), Lymph node (−),N = 228*	0.5	**0.037**
*ER(−), PR(−), N = 298*	0.74	0.14
*ER(−), PR(−), Lymph node(+),N = 127*	0.76	0.3
*ER(−), PR(−), Lymph node(−),N = 167*	0.97	0.93
*ER(−), PR(−), HER2(−),N = 198*	0.93	0.77
*Basal subtype, N = 618*	0.68	**0.0025**
*Luminal A, N = 1933*	0.76	**0.0015**
*Luminal B, N = 1149*	0.77	**0.0069**
*ER(+), HER2(+), N = 156*	1.21	0.53
*ER(−), HER2(+), N = 96*	0.61	0.12
**TGR5 (1552501_at)**	**HR (Hazard Ratio)**	***p*-Value (Log Rank Test)**
*All Breast Cancers, N = 3951*	0.91	0.25
*Grade 1, N = 345*	0.41	0.12
*Grade 2, N = 901*	1.21	0.46
*Grade 3, N = 903*	0.77	0.1
*ER(+), N = 3082*	1	0.98
*ER(−), N = 869*	0.74	**0.025**
*PR(+), N = 589*	1.21	0.33
*PR(−), N = 549*	0.96	0.81
*Lymph(+), N = 1133*	0.98	0.9
*Lymph(−), N = 2020*	0.91	0.61
*HER2(+), N = 252*	1.12	0.68
*HER2(−), N = 800*	0.91	0.51
*ER(+), PR(+), N = 577*	1.18	0.4
*ER(+), PR(+), Lymph node(+),N = 344*	1.39	0.19
*ER(+), PR(+), Lymph node(−),N = 228*	0.76	0.4
*ER(−), PR(−), N = 298*	0.96	0.86
*ER(−), PR(−), Lymph node(+),N = 127*	0.96	0.89
*ER(−), PR(−), Lymph node(−),N = 167*	1.25	0.61
*ER(−), PR(−), HER2(−),N = 198*	0.9	0.73
*Basal subtype, N = 618*	0.79	0.15
*Luminal A, N = 1933*	1.1	0.44
*Luminal B, N = 1149*	0.83	0.24
*ER(+), HER2(+), N = 156*	1.07	0.88
*ER(−), HER2(+), N = 96*	0.94	0.86
**NRF2 (201145_at)**	**HR (Hazard Ratio)**	***p*-Value (Log Rank Test)**
*All Breast Cancers, N = 3951*	1.23	**2.00 × 10^−4^**
*Grade 1, N = 345*	1.1	0.72
*Grade 2, N = 901*	0.87	0.25
*Grade 3, N = 903*	1.06	0.63
*ER(+), N = 3082*	1.27	**2.40 × 10^−4^**
*ER(−), N = 869*	1.04	0.71
*PR(+), N = 589*	1.23	0.24
*PR(−), N = 549*	1.49	**0.0075**
*Lymph(+), N = 1133*	1.14	0.21
*Lymph(−), N = 2020*	1.11	0.21
*HER2(+), N = 252*	1.32	0.21
*HER2(−), N = 800*	1.34	**0.031**
*ER(+), PR(+), N = 577*	1.27	0.19
*ER(+), PR(+), Lymph node(+),N = 344*	1.08	0.71
*ER(+), PR(+), Lymph node(−),N = 228*	1.96	0.051
*ER(−), PR(−), N = 298*	1.01	0.95
*ER(−), PR(−), Lymph node(+),N = 127*	1.23	0.45
*ER(−), PR(−), Lymph node(−),N = 167*	0.93	0.8
*ER(−), PR(−), HER2(−),N = 198*	0.9	0.66
*Basal subtype, N = 618*	1.01	0.95
*Luminal A, N = 1933*	1.33	**0.0011**
*Luminal B, N = 1149*	1.26	**0.017**
*ER(+), HER2(+), N = 156*	1.69	0.097
*ER(−), HER2(+), N = 96*	1.26	0.47
**KEAP1 (202417_at)**	**HR (Hazard Ratio)**	***p*-Value (Log Rank Test)**
*All Breast Cancers, N = 3951*	0.84	**0.0015**
*Grade 1, N = 345*	0.98	0.94
*Grade 2, N = 901*	0.73	**0.012**
*Grade 3, N = 903*	0.93	0.54
*ER(+), N = 3082*	0.89	0.06
*ER(−), N = 869*	0.92	0.41
*PR(+), N = 589*	1.09	0.64
*PR(−), N = 549*	0.91	0.55
*Lymph(+), N = 1133*	0.76	**0.006**
*Lymph(−), N = 2020*	0.98	0.83
*HER2(+), N = 252*	1.14	0.57
*HER2(−), N = 800*	0.92	0.55
*ER(+), PR(+), N = 577*	1.06	0.76
*ER(+), PR(+), Lymph node(+),N = 344*	0.98	0.91
*ER(+), PR(+), Lymph node(−),N = 228*	1.38	0.33
*ER(−), PR(−), N = 298*	0.95	0.82
*ER(−), PR(−), Lymph node(+),N = 127*	1	1
*ER(−), PR(−), Lymph node(−),N = 167*	1.12	0.72
*ER(−), PR(−), HER2(−),N = 198*	1.07	0.8
*Basal subtype, N = 618*	1	0.98
*Luminal A, N = 1933*	0.9	0.24
*Luminal B, N = 1149*	0.91	0.36
*ER(+), HER2(+), N = 156*	0.89	0.7
*ER(−), HER2(+), N = 96*	1.18	0.61
**iNOS (210037_at)**	**HR (Hazard Ratio)**	***p*-Value (Log Rank Test)**
*All Breast Cancers, N = 3951*	0.72	**5.40 × 10^−9^**
*Grade 1, N = 345*	1.21	0.48
*Grade 2, N = 901*	0.95	0.68
*Grade 3, N = 903*	0.85	0.15
*ER(+), N = 3082*	0.8	**0.00066**
*ER(−), N = 869*	0.63	**1.40 × 10^−5^**
*PR(+), N = 589*	1.34	0.099
*PR(−), N = 549*	1.01	0.95
*Lymph(+), N = 1133*	1.12	0.25
*Lymph(−), N = 2020*	1	0.96
*HER2(+), N = 252*	0.79	0.3
*HER2(−), N = 800*	1.04	0.75
*ER(+), PR(+), N = 577*	1.32	0.13
*ER(+), PR(+), Lymph node(+),N = 344*	1.52	0.058
*ER(+), PR(+), Lymph node(−),N = 228*	1.04	0.9
*ER(−), PR(−), N = 298*	0.90	0.59
*ER(−), PR(−), Lymph node(+),N = 127*	1.03	0.91
*ER(−), PR(−), Lymph node(−),N = 167*	0.91	0.77
*ER(−), PR(−), HER2(−),N = 198*	0.8	0.37
*Basal subtype, N = 618*	0.63	**0.00042**
*Luminal A, N = 1933*	0.75	**0.00082**
*Luminal B, N = 1149*	0.77	**0.0077**
*ER(+), HER2(+), N = 156*	1.04	0.9
*ER(−), HER2(+), N = 96*	0.92	0.79
**nNOS (207309_at)**	**HR (Hazard Ratio)**	***p*-value (Log Rank Test)**
*All Breast Cancers, N = 3951*	0.71	**6.70 × 10^−10^**
*Grade 1, N = 345*	0.85	0.55
*Grade 2, N = 901*	0.99	0.96
*Grade 3, N = 903*	0.91	0.41
*ER(+), N = 3082*	0.77	**5.80 × 10^−5^**
*ER(−), N = 869*	0.68	**2.80 × 10^−4^**
*PR(+), N = 589*	0.79	0.19
*PR(−), N = 549*	1.07	0.67
*Lymph(+), N = 1133*	1.08	0.47
*Lymph(−), N = 2020*	0.91	0.25
*HER2(+), N = 252*	0.7	0.11
*HER2(−), N = 800*	0.92	0.51
*ER(+), PR(+), N = 577*	0.86	0.41
*ER(+), PR(+), Lymph node(+),N = 344*	1.23	0.35
*ER(+), PR(+), Lymph node(−),N = 228*	0.34	**0.0055**
*ER(−), PR(−), N = 298*	0.96	0.83
*ER(−), PR(−), Lymph node(+),N = 127*	1.07	0.8
*ER(−), PR(−), Lymph node(−),N = 167*	1.55	0.16
*ER(−), PR(−), HER2(−),N = 198*	1.17	0.53
*Basal subtype, N = 618*	0.73	**0.013**
*Luminal A, N = 1933*	0.72	**0.00019**
*Luminal B, N = 1149*	0.69	**0.00014**
*ER(+), HER2(+), N = 156*	1.4	0.28
*ER(−), HER2(+), N = 96*	0.48	0.024
**nNOS (207310_at)**	**HR (Hazard Ratio)**	***p*-Value (Log Rank Test)**
*All Breast Cancers, N = 3951*	0.8	**3.80 × 10^−5^**
*Grade 1, N = 345*	1.27	0.37
*Grade 2, N = 901*	1.20	0.14
*Grade 3, N = 903*	0.89	0.27
*ER(+), N = 3082*	0.84	**7.70 × 10^−3^**
*ER(−), N = 869*	0.72	**1.90 × 10^−3^**
*PR(+), N = 589*	1.07	0.72
*PR(−), N = 549*	1.04	0.8
*Lymph(+), N = 1133*	1	0.97
*Lymph(−), N = 2020*	1.09	0.34
*HER2(+), N = 252*	0.78	0.26
*HER2(−), N = 800*	1	0.97
*ER(+), PR(+), N = 577*	1.07	0.73
*ER(+), PR(+), Lymph node(+),N = 344*	1.51	0.06
*ER(+), PR(+), Lymph node(−),N = 228*	0.59	0.13
*ER(−), PR(−), N = 298*	1.18	0.41
*ER(−), PR(−), Lymph node(+),N = 127*	1.02	0.93
*ER(−), PR(−), Lymph node(−),N = 167*	1.42	0.25
*ER(−), PR(−), HER2(−),N = 198*	1.44	0.14
*Basal subtype, N = 618*	0.76	**0.037**
*Luminal A, N = 1933*	0.75	**0.0011**
*Luminal B, N = 1149*	0.85	0.09
*ER(+), HER2(+), N = 156*	1.15	0.65
*ER(−), HER2(+), N = 96*	0.45	**0.013**
**NOX4 (236843_at)**	**HR (Hazard Ratio)**	***p*-Value (Log Rank Test)**
*All Breast Cancers, N = 3951*	0.75	**0.00027**
*Grade 1, N = 345*	0.76	0.6
*Grade 2, N = 901*	0.68	0.14
*Grade 3, N = 903*	0.69	0.79
*ER(+), N = 3082*	0.7	**0.00027**
*ER(−), N = 869*	0.8	0.1
*PR(+), N = 589*	0.71	0.082
*PR(−), N = 549*	1.04	0.85
*Lymph(+), N = 1133*	0.78	0.057
*Lymph(−), N = 2020*	1.08	0.71
*HER2(+), N = 252*	1.31	0.33
*HER2(−), N = 800*	0.81	0.16
*ER(+), PR(+), N = 577*	0.69	0.06
*ER(+), PR(+), Lymph node(+),N = 344*	0.73	0.21
*ER(+), PR(+), Lymph node(−),N = 228*	0.97	0.93
*ER(−), PR(−), N = 298*	1.23	0.41
*ER(−), PR(−), Lymph node(+),N = 127*	1.35	0.34
*ER(−), PR(−), Lymph node(−),N = 167*	1.07	0.87
*ER(−), PR(−), HER2(−),N = 198*	1.25	0.49
*Basal subtype, N = 618*	0.72	**0.046**
*Luminal A, N = 1933*	0.71	**0.0061**
*Luminal B, N = 1149*	0.77	0.089
*ER(+), HER2(+), N = 156*	1.34	0.51
*ER(−), HER2(+), N = 96*	1.5	0.25

**Table 5:** Numerical values for kmplot analysis in Figure 7.

Probe				Time (Months)						HR
				0	50	100	150	200	250	
**All cancers**	**Number at risk**	**CAR**	low	1998	1154	486	124	10	2	0.7 (0.63–0.79)
		207007_at	high	1953	1365	589	117	17	1	
		**TGR5**	low	58	41	13	1			0.41 (0.13–1.3)
		1552501_a_at	high	50	44	15	4			
		**NRF2**	low	1977	1368	624	145	11	0	1.23 (1.1–1.37)
		201146_at	high	1974	1151	451	96	16	3	
		**KEAP1**	low	1978	1181	513	131	17	2	0.84 (0.75–0.93)
		202417_at	high	1973	1338	562	110	10	1	
		**iNOS**	low	1993	1154	446	107	14	2	0.72 (0.65–0.81)
		210037_s_at	high	1958	1365	629	134	13	1	
		**nNOS**	low	1981	1128	454	114	14	3	0.71 (0.64–0.79)
		207309_at	high	1970	1391	621	127	13	0	
		**nNOS**	low	2016	1185	470	112	15	2	0.8 (0.71–0.89)
		207310_s_at	high	1935	1334	605	129	12	1	
		**NOX4**	low	885	464	145	32	6	1	0.75 (0.64–0.88)
		236843_at	high	879	513	200	36	4	1	
**ER +**	**Number at risk**	**CAR**	low	1561	1024	443	114	12	3	0.81 (0.71–0.92)
		207007_at	high	1521	1092	476	87	10	0	
		**TGR5**	low	628	382	149	25	5	1	1 (0.83–1.22)
		1552501_a_at	high	620	399	139	35	5	1	
		**NRF2**	low	1541	1132	525	113	6	0	1.27 (1.12–1.44)
		201146_at	high	1541	984	394	88	16	3	
		**KEAP1**	low	1544	1011	448	106	14	2	0.89 (0.78–1.01)
		202417_at	high	1538	1105	471	95	8	1	
		**iNOS**	low	1543	995	393	97	14	2	0.8 (0.71–0.91)
		210037_s_at	high	1539	1121	526	104	8	1	
		**nNOS**	low	1544	986	407	105	17	3	0.77 (0.68–0.88)
		207309_at	high	1538	1130	512	96	5	0	
		**nNOS**	low	1561	1013	409	101	12	2	0.84 (0.74–0.96)
		207310_s_at	high	1521	1103	510	100	10	1	
		**NOX4**	low	639	387	129	28	6	1	0.7 (0.57–0.85)
		236843_at	high	609	394	159	32	4	1	
**Triple negative**	**Number at risk**	**CAR**	low	99	42	13	1	0		0.93 (0.57–1.51)
		207007_at	high	99	41	6	1	0		
		**TGR5**	low	64	30	8	1	0		0.9 (0.48–1.68)
		1552501_a_at	high	62	29	9	1	0		
		**NRF2**	low	99	41	5	1	0		0.9 (0.55–1.45)
		201146_at	high	99	42	14	1	0		
		**KEAP1**	low	99	44	12	2	0		1.07 (0.66–1.73)
		202417_at	high	99	39	7	0	0		
		**iNOS**	low	99	44	15	1	0		0.8 (0.49–1.3)
		210037_s_at	high	99	39	4	1	0		
		**nNOS**	low	100	44	16	1	0		1.77 (0.72–1.9)
		207309_at	high	98	39	3	1	0		
		**nNOS**	low	102	51	14	2	0		1.44 (0.89–2.35)
		207310_s_at	high	96	32	5	0	0		
		**NOX4**	low	64	30	8	2	0		1.25 (0.66–2.35)
		236843_at	high	62	29	9	0	0		

## Data Availability

All primary data is uploaded to https://figshare.com/s/e4ac81e7d47f23aea58f (DOI: 10.6084/m9.figshare.7541975).

## Abbreviations

CAR	constitutive androstane receptor
CAT	catalase
CA	cholic acid
CDCA	chenodeoxycholic acid
ER	estrogen receptor
FXR	farnesoid X-activated receptor
GCLC	glutamate–cysteine ligase catalytic subunit
GSH	reduced glutathione
GPX2	glutathione peroxidase 2
GPX3	glutathione peroxidase 3
Her2	Human epidermal growth factor receptor 2/herceptin receptor/erbB receptor
HMOX1	heme oxygenase 1
iNOS	inducible NO synthase
KEAP1	Kelch like ECH associating protein 1
LCA	lithocholic acid
LXR	liver X nuclear receptor
Mib1	molecular immunology borstel-1
NOX4	NADPH oxidase 4
NAC	N-acetylcysteine
nNOS	neuronal NOS
NQO1	NAD(P)H quinone dehydrogenase 1
NRF2	nuclear factor, erythroid 2-like 2
PR	progesterone receptor
PXR	pregnane X receptor
SOD1	superoxide dismutase 1
SOD2	superoxide dismutase 2
SOD3	superoxide dismutase 3
SRB	sulphorhodamine B
TBA	thiobarbituric acid
TBARS	thiobarbituric acid reactive substances
TGR5/GPBAR1	G protein-coupled bile acid receptor 1/Takeda G-protein coupled receptor
TNBC	triple negative breast cancer
VDR	vitamin D receptor
4HNE	4-hydroxynonenal
